# Exploring In Vivo Models of Musculoskeletal Frailty: A Comprehensive Systematic Review

**DOI:** 10.3390/ijms242316948

**Published:** 2023-11-29

**Authors:** Deyanira Contartese, Laura Di Sarno, Francesca Salamanna, Lucia Martini, Milena Fini, Gianluca Giavaresi, Francesca Veronesi

**Affiliations:** 1 Surgical Sciences and Technologies, IRCCS Istituto Ortopedico Rizzoli, Via di Barbiano 1/10, 40136 Bologna, Italy; deyanira.contartese@ior.it (D.C.); francesca.salamanna@ior.it (F.S.); lucia.martini@ior.it (L.M.); gianluca.giavaresi@ior.it (G.G.); francesca.veronesi@ior.it (F.V.); 2 Scientific Direction, IRCCS Istituto Ortopedico Rizzoli, Via di Barbiano 1/10, 40136 Bologna, Italy; milena.fini@ior.it

**Keywords:** frailty, musculoskeletal system, in vivo models, systematic review

## Abstract

Musculoskeletal frailty—a common and debilitating condition linked to aging and chronic diseases—presents a major public health issue. In vivo models have become a key tool for researchers as they investigate the condition’s underlying mechanisms and develop effective interventions. This systematic review examines the current body of research on in vivo models of musculoskeletal frailty, without any time constraints. To achieve this aim, we utilized three electronic databases and incorporated a total of 11 studies. Our investigation delves into varied animal models that simulate specific features of musculoskeletal frailty, including muscle loss, bone density reduction, and functional decline. Furthermore, we examine the translational prospects of these models in augmenting our comprehension of musculoskeletal frailty and streamlining the production of groundbreaking therapeutic approaches. This review provides significant insights and guidance for healthcare researchers and practitioners who aim to combat musculoskeletal frailty, ultimately enhancing the quality of life for older adults and individuals affected by this condition.

## 1. Introduction

The definition of the frail phenotype was first given in the geriatric literature by Fried in 2001 [1], and in 2012, the consensus conference led by the International Association of Gerontology and Geriatrics and the World Health Organization defined frailty as “a medical syndrome with multiple causes and contributors, characterized by decreased strength, endurance and physiological function that increases an individual’s vulnerability to developing increased dependency and/or death” [2]. Frailty is a biological syndrome characterized by a multi-systemic decline, particularly in physical function, accompanied by significant inflammation (known as “inflammaging”) and an increased risk of hospitalization, disability, and death [3,4]. Frailty is highly vulnerable to low-pressure stressors, resulting in decreased functional resilience, cumulative decline in multiple body systems, and multi-organ dysfunctions [2]. Frailty is linked to oxidative stress that disturbs the redox signaling balance found in healthy physiology and results in enduring harm to cellular functions and homeostasis, as per the “free radical theory of frailty” [5]. It is projected that the number of frail individuals will rise over the next three decades due to increased life expectancy. Although frailty does increase with age (4–26% from 65 to 85 years) [6], it is not always linked to aging, as some senior citizens remain vigorous [7]. It is important to note that frailty is a complex and multidimensional concept, and its presence or absence depends on a combination of biological, psychological, social, and environmental factors. Furthermore, frailty can vary over time and can be managed or prevented through appropriate interventions, such as exercise programs, lifestyle modifications, and appropriate medical care.

Concerning the musculoskeletal system, physical performance in frail individuals decreases as morbidity and mortality rates increase [7]. Weakness that results in osteopenia and sarcopenia is a factor in frailty. A decrease in muscle mass, movement capacity, and other physical functions is evident, which leads to a higher chance of falling, disability, fractures, and mortality [8,9,10]. In addition, the intramuscular connective tissue plays a critical role in maintaining the structural integrity of the muscle and in providing mechanical support during aging. It was observed that the accumulation of collagen I and the reduction in hyaluronan and elastic fibers with aging could cause stiffening and adaptability reduction in the muscles [11,12,13].

Various tools have been used to assess frailty, such as the accumulation of deficits and frailty phenotypes, and specific tools have been developed, so there is no consensus [14]. Frailty indices are the most frequently used measure of health deficits accompanying aging, with several items ranging from 5 to over 30, and are calculated by dividing the number of deficits by their total [15].

There are numerous resemblances in the behavioral frailty indices (FIs) between humans and mice. As a result, mice are used in in vivo studies to comprehend frailty and frailty interventions [16,17,18,19]. The FI provides longitudinal, non-invasive measures that can be utilized in preclinical models to assess general health status. Consequently, animal models of frailty have been increasing lately, since the build-up of health deficits can be assessed over time.

Given the popularity of the topic of frailty in recent years, this systematic review collects and analyses all of the in vivo studies, without time limits, performed on animal models of musculoskeletal frailty. The review discusses the employed models, the evaluation tools used for musculoskeletal frailty, and information relating to therapeutic strategies. No other systematic review evaluates in vivo models set up to recreate musculoskeletal frailty.

## 2. Materials and Methods

### 2.1. Eligibility Criteria

A PICO question (Population of interest (P), Intervention (I), Comparators and Outcomes (CO)) was employed to select and analyze the relevant papers. The “Population” considered was in vivo models of musculoskeletal system frailty. The “Intervention” was an all-in-vivo intervention through which frailty was induced. The “Comparator”, when available, was healthy animals or animals treated with a potential therapeutic strategy. The primary “Outcome” was musculoskeletal frailty evaluation through different techniques. In addition, a secondary “Outcome” was the effect of some treatments used to reduce frailty when present.

### 2.2. Search Strategy

**Figure 1:**
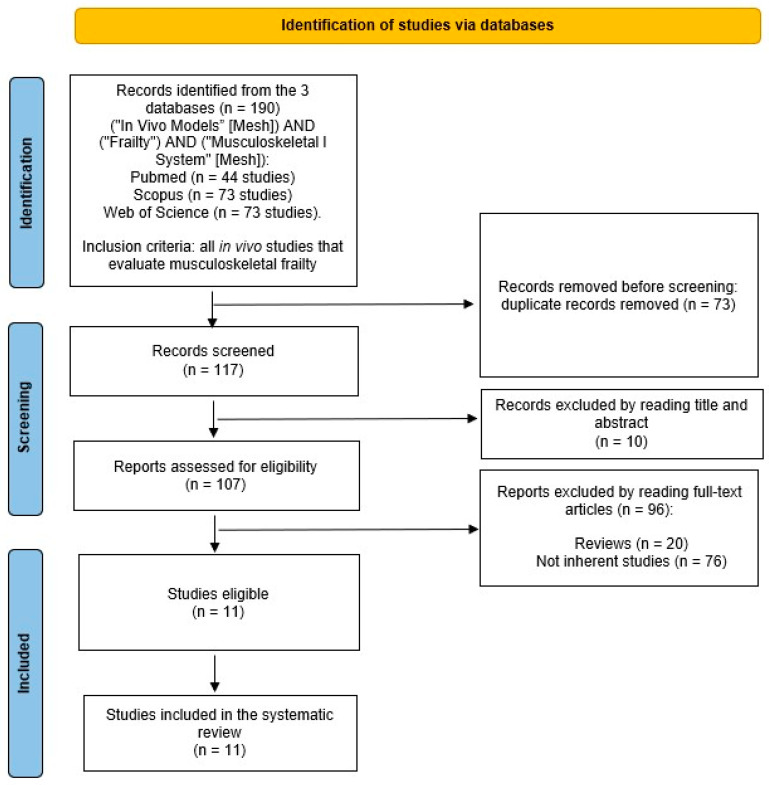
Flowchart of the included studies according to PRISMA principles.

The search was performed on 29 September 2023, and included research published every year, without publication year limits, according to the Preferred Reporting Items for Systematic Reviews and Meta-Analyses (PRISMA) statement (Figure 1).

To identify relevant papers, we used three electronic databases (PubMed, Scopus, and Web of Science) and the following MeSH: (“In Vivo Models” (MeSH)) AND (“Frailty”) AND (“Musculoskeletal l System” (MeSH)). The limit was the English language.

After removing duplicates (Mendeley 1.14, www.mendeley.com; accessed on 29 September 2023), two authors (FV and DC) screened relevant articles using title and abstract. Excluded were articles that did not satisfy the inclusion criteria. The two authors then retrieved and scrutinized the complete texts of the included articles, reconciling any disputes through discussion until consensus was achieved, or with the interference of a third author (LDS). The information extracted from each paper summarizes the evidence reported in each study, including an in vivo model of frailty, evaluations, main results, and references (Ref.) (Table 1).

### 2.3. Risk of Bias Assessment

Two reviewers (FV and DC) individually analyzed the methodological quality of the included studies. In cases of disagreement, they tried to reach consensus; if this failed, a third reviewer (LDS) made the definitive decision. The methodological quality assessment of the included in vivo studies was carried out according to the Systematic Review Centre for Laboratory Animal Experimentation (SYRCLE) tool [31], which has been developed to assess the risk of bias in animal studies.

## 3. Results

The search for literature yielded a total of 190 studies, comprising 44 studies from PubMed, 73 studies from Scopus, and 73 papers from the Web of Science. Following the elimination of duplicates (73 papers), 117 papers were subjected to screening, and another 10 papers were excluded. A total of 96 articles were excluded after reading them in full because they were not original studies (n = 76) or reviews (n = 20). Finally, the systematic review included 11 eligible articles (Figure 1). All studies examined frailty and possible treatments in an in vivo mouse model of frailty, using different methods: (1) elderly models; (2) genetically modified animals; (3) injection of peptide or cardiotoxin; and (4) tail suspension (TS). The results are summarized in Table 1, and the items of the FI employed in these studies are listed in Table 2.

### 3.1. In Vivo Models of Frailty

#### 3.1.1. Elderly Models

Five studies utilized the mouse models C57BL/6J [20,21,24], C57BL/6N [22], and C57BL/6 [23] at different age ranges.

In their research, Asadi Shahmirzadi et al. investigated the effects of a diet containing 2% *w*/*w* alpha-ketoglutarate (AKG) compared to a standard diet in mice that were both 18 months old and deemed frail by comparison to younger adults [20]. No young mice were included as a control group. The evaluation of food intake, survival, indirect calorimetry, and treadmill exhaustion test indicated that survival and lifespan were notably reduced, whereas FI, O_2_ consumption, and CO_2_ production were substantially higher in the mice fed with a standard diet as opposed to those fed with AKG [20]. Mice at 6 (young), 24 (adult), and >28 (old) months of age were evaluated with functional tests and body, muscle mass, and muscle contractile physiology evaluations. It was observed that grip, CFAB score, and muscle contractile physiology were lower and muscle mass was higher in 24- and >28-month-old mice than in younger ones. In addition, voluntary wheel running was significantly lower in mice >28 months of age than in the younger two groups [21].

Similarly, Petr et al. evaluated the FI, body composition, functional tests, tibia micro-computed tomography (Micro-CT), metabolic assessment, and O_2_ consumption in mice with three ranges of age (3–8, 13–23, and 27–36 months). The oldest group of animals showed higher FI, respirator exchange ratio (RER), and lean mass around tibia percentage and lower gait speed, forelimb muscle strength, locomotor activity, cortical thickness (Cr.Th) and bone mineral density (BMD), body weight, and fat around tibia percentage in comparison with the other two groups [22].

Arc-Chagnaud et al. employed old (18–26 months of age) and very old (34 months of age) wild-type (WT) mice. In addition, glucose 6-P dehydrogenase (G6PD)-overexpressing (G6PD-Tg) mice, that are protected against metabolic stresses, were compared. The frailty score and RER were significantly lower, while locomotory activity was significantly higher in the G6PD-Tg mice of 18–26 months of age in comparison to the WT. In addition, muscle fiber size was significantly higher in the G6PD-Tg mice than in WT ones at 34 months of age [23].

Finally, two treatments—MyMD-1, a synthetic derivative of the alkaloid myosmine, and rapamycin, administered through the water to drink—were compared in adult mice (19 months of age) [24]. Health span and lifespan assessments were made and FI was evaluated. Although body weight and muscle strength decreased and FI increased with age in all treatment groups, MyMD-1 induced higher health span characteristics, muscle strength, and survival and lower body weight loss and FI in comparison to rapamycin treatment at both high and low doses [24].

#### 3.1.2. Genetically Modified Animal Model

In a further three studies, the authors set up in vivo models of frailty in genetically modified mice [25,26,27].

Akki et al. employed the homozygous interleukin-10 null, B6.129P2-IL10™/Cgn/J (IL10™/™) mice, a validated animal model of frailty, at the age of 92 weeks and compared them with C57BL/6J mice of the same age. The authors focused on sarcopenia of the hind limb skeletal muscle, through 31P magnetic resonance spectroscopy (MRS), rate of adenosine triphosphate (ATP) synthesis from inorganic phosphate (Pi), and free energy released from ATP hydrolysis, showing that phosphocreatine, ATP flux via creatine kinase (CK), ATP synthesis from inorganic phosphate, and the free energy released from ATP hydrolysis were lower and Pi higher in the genetically modified animals [25].

Scheuren et al. used PolgA^(D257A/D257A)^ mutator mice, at the age ranging from 20 to 46 weeks, as a model of frailty and senile osteoporosis [26,27]. The authors evaluated FI and bone remodeling activity through static and dynamic Micro-CT of the femur and 6th caudal vertebrae, and conducted functional tests. In comparison to the control PolgA^(+/+)^ mice, PolgA^(D257A/D257A)^ ones exhibited higher health deficits and lower bone and muscle mass and functionality and bone remodeling activities, at all ages, and higher FI score and lower grip strength at 40 and 46 weeks of age [26]. In addition, it was also observed that bone volume/tissue volume (BV/TV) and trabecular thickness (Tb.Th) decreased in both control and PolgA^(D257A/D257A)^ mice during aging [27].

#### 3.1.3. Peptide Injection Animal Model

In two studies, animal models of frailty were induced through the injection of cardiotoxin (CTX) into the tibialis anterior or quadriceps muscles [28], or of Myelin Oligodendrocyte Glycoprotein (MOG) peptide emulsified in Complete Freud’s Adjuvant (CFA) supplemented with heat-inactivated Mycobacterium tuberculosis, subcutaneously [29], in C57BL/6J [29] and C57BL/6 [29] mice.

Jing et al. carried out functional tests in mice of 16 months old, showing that grip strength, physical endurance, and maximal running time and distance were significantly lower in the CTX-injected mice in comparison to sham mice or mice treated with recombinant Sestrin 1 (rSESN1) protein [28].

Ribeiro et al. showed that the injection of MOG peptide, emulsified in CFA supplemented with heat-inactivated Mycobacterium tuberculosis, in mice at 3 months of age induced lower body weight and higher FI, which further increased in mice aged 12 months in comparison to those at 3 and 6 months [29].

#### 3.1.4. Tail Suspension Animal Model

Ono et al. performed another model of frailty by using the TS procedure to mimic disuse-induced frailty in C57BL/6J mice of 6 weeks of age. Micro-CT of the femur, histology of the proximal tibia and of soleus muscle, histomorphometry, and functional tests were performed. The TS model induced more of a reduction in muscle fiber width, travel distance, maximal muscle strength, BV/TV, trabecular number and thickness, bone mineral content/tissue volume, Cr.Th., bone formation rate, osteoblast surface, bone mass, and osteoid surface and more of an increase in episodes of fatigue-like behavior, osteoclast number, and eroded surface in comparison to mice of the same model treated with locamidazole (LAMZ). No comparison with mice who did not undergo TS was made [30].

### 3.2. Gender Differences

Two studies assessed the differences between male and female mice [20,24]. Frail mice administered with MyMD-1 and rapamycin exhibited reduced body weight in females compared to males (*p* < 0.001). Moreover, frailty increased with age for both sexes, but mainly affected females (*p* = 0.02). Lastly, male mice had significantly higher survival rates and lifespan compared to females (*p* < 0.0001 and *p* < 0.001, respectively) [24]. In contrast, one study found no disparities between male and female mice across all assessed parameters. However, amongst mice fed AKG, female survival rates were significantly higher (*p* = 0.037), while male survival rates did not yield statistically significant findings [20]. Notably, the remaining investigations exclusively utilized one gender of animals.

### 3.3. Risk of Bias Assessment

**Figure 2:**
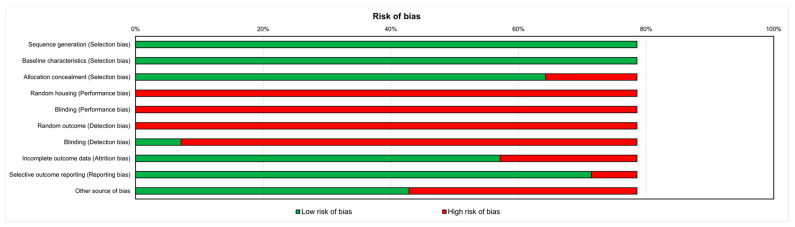
Results of risk of bias of the included studies.

Figure 2 shows that there was a high risk of bias for nearly all of the papers. All in vivo studies included indicated the method of sequence generation and showed that the groups had comparable baseline characteristics. Approximately 75% of the studies concealed the allocation adequately, while two studies did not. There was no employment of random housing, housed blinding, or random outcome assessment during the experiment. Only one study selected assessor blinding. Almost all of the studies analyzed included all animals (n = 8), had specified primary outcomes (n = 10), and were devoid of other biases that might have caused a high risk (n = 6).

## 4. Discussion

The current systematic review collects all of the literature pertaining to in vivo models of musculoskeletal frailty and potential therapeutic approaches that can delay the progression of frailty or alleviate the adverse effects of frailty on the musculoskeletal system. Additionally, this systematic review highlights the methods employed by the authors for identifying and assessing musculoskeletal frailty.

This review solely focuses on musculoskeletal frailty rather than aging animal models. Frailty has become an increasingly popular topic. It is defined as a decrease in physiological function, and it is separate from the concept of aging. In fact, physiological aging is distinct from chronological aging [32]. In addition, frailty acts as a predictor of morbidity and mortality in elderly patients, more so than age alone [33].

This phenomenon is the result of several interrelated mechanisms and processes. Some of the major factors involved in musculoskeletal frailty include sarcopenia, osteopenia and osteoporosis, connective tissue changes, and chronic inflammation [34].

Only 11 in vivo studies explore musculoskeletal frailty, a topic that receives relatively limited attention in the vast literature on aging. In comparison to frailty found in other organs and systems, such as the brain, the findings available on the musculoskeletal system are scarce. The studies collected are recent, with three performed in 2020 [20,26,27], three in 2021 [21,22,23], two in 2022 [29,30], and a further two in 2023 [24,28]. Only one study was published in 2014 [26]. The models employed to induce frailty in vivo differ, with mice being the preferred animal model. Mice under controlled conditions can live for up to 4 years [35], displaying several histopathological features comparable to those of human aging, including tissue inflammation, necrosis, and cancer [36].

In the studies included in this review, all mice were old, but most of the frailty assessments were only evaluated in adult and old mice (5/11 studies) [20,21,22,23,24], since frailty occurs mainly in the elderly [6]. In the other studies, frailty was evaluated in genetically modified animals (n = 3), after peptide injections (n = 2), and in a TS model (n = 1).

In two studies, three groups of mice with three different age ranges were compared in terms of musculoskeletal frailty: young (3–8 months), adult (13–24 months), and old (27–36 months) [22,23]. In adult and old mice, grip strength, CFAB score, muscle contractile physiology, and voluntary wheel running were observed to be lower in comparison to young mice. The frailty characteristics further increased in old mice with higher RER and lean tissue percentage and lower gait speed, locomotor activity, and BMD in comparison to young and adult animals [22,23].

Once the presence of frailty had been established in adult and old mice, treatments against frailty were tested [20,23,24]. The addition of AKG to the standard diet increased survival and lifespan and reduced FI, O2 consumption, CO2 production, and energy expenditure [20]. Indeed, AKG, an inductor of the anti-inflammatory cytokine interleukin 10 (IL10), has been observed to be involved in various fundamental processes, including central metabolism, collagen synthesis, epigenetic regulation, and stem cell proliferation [37,38]. In addition, MyMD-1, capable of suppressing tumor necrosis factor alpha (TNF-α) production [39], was compared to rapamycin, the best characterized drug endowed with antiaging properties due to its autophagy suppressor, immunosuppressive, anti-inflammatory, and antiproliferation effects [40]. MyMD-1 induced higher health span, muscle strength, and survival and lower frailty and weight loss in comparison to rapamycin [24]. Furthermore, the frailty score and RER decreased and locomotory activity and muscle fiber size increased more in the G6PD-Tg mice than in the WT [23]. In the literature, G6PD-Tg mice were found to be less affected by ROS-derived damage and very protected from metabolic stresses [41], since the overexpression of G6PD leads to higher levels of NADPH and lower levels of ROS-derived damage concomitant with an extended lifespan [42].

Another murine frailty model was performed using genetically modified animals (3/11 studies) [25,26,27].

PolgA^(D257A/D257A)^ mice, used in two studies [26,27], exhibited an accelerated aging phenotype (such as hair loss, graying, and hearing loss) due to elevated mitochondrial DNA point mutations and systemic mitochondrial dysfunction in comparison to the PolgA^(+/+)^ WT mice [43,44]. In the present review, PolgA^(D257A/D257A)^ mice exhibited higher health deficits and FI and lower bone remodeling activity, grip strength, and concentric muscle forces [26,27].

In addition, IL10™/™ mice, characterized by a high level of serum IL6, muscle weakness, and high mortality [45,46], were employed as a model of sarcopenia because ATP kinetics, high-energy phosphate levels, and energy release from ATP hydrolysis were reduced and inorganic phosphate was increased [25].

Then, the injection of CTX into the tibialis anterior or quadriceps muscles [28], and of MOG peptide, emulsified in CFA supplemented with heat-inactivated Mycobacterium tuberculosis, in the subcutis [29] induced frailty (2/11 studies).

The injection of CTX and of MOG peptide reduced grip strength, physical endurance, and maximal running time and distance [28], and increased frailty [29]. The injection of MOG peptide is a model of autoimmune encephalomyelitis used to study multiple sclerosis. It is observed that middle-aged individuals with multiple sclerosis have mobility deficits like those seen in much older individuals without multiple sclerosis [47].

With regard to therapeutic strategies, the administration, in this last animal model, of rSESN1, a metabolism protein induced in cells by oxidative stress, DNA damage, hypoxia, and starvation [48], increased the grip strength, physical endurance, and maximal running time and distance in frail mice [28]. Previous studies showed that this protein protected against metabolism disorders, lipid accumulation, and insulin resistance [49].

The last frailty model adopted was the TS model (1/11 study) [30], in which the tail is suspended, thus preventing the hindlimb from resting on the floor, mimicking disuse-induced frailty. The authors evaluated the effects of LAMZ, an aminoindazole derivative, on frailty, showing that mice treated with LAMZ increased muscle fiber width, travel distance, muscle strength, bone formation, and reduced episodes of fatigue and bone resorption [30].

Another factor underlined by the present review was the different ways, used by the authors, for the evaluations of the level of musculoskeletal frailty in the above-mentioned models. They are grouped into functional tests [20,22,23,26,28,29], body composition and muscle mass measures [22,23], micro-CT bone parameters [22,26,27,30], bone and muscle histology, immunohistochemistry (IHC) and histomorphometry [23,30], indirect calorimetry [20,22], metabolic assessment [20,23], survival and health span assessment [20,24], and FI [20,21,22,23,24,26,27,29].

Among them, FIs deserve a broader discussion, because they are usually employed to evaluate frailty in humans. These indices are composed of items that comprise the presence of some concomitant pathologies, functional status, mood, cognitive capacity, and health deficits as measured by a physician or by the patient themselves. The cut-off that stratifies the patients is obtained by dividing the number of positive items by the total number of items [50]. However, more than 20 different indices are used to measure frailty, with no consensus on the most appropriate one being reached [51].

In the present review, to evaluate frailty, in a similar manner to humans, some human FIs were readapted to the mouse models. The approach used most often (4/7 studies) was the mouse frailty assessment designed by Whitehead et al. [20,22,26,27]. It is noninvasive index that is simple to implement, based on 31 items, that provides a robust estimation of frailty that is also able to be translated into humans [16]. The 31 items are grouped into “Integument”, “Physical/musculoskeletal”, “Vestibulocochlear/auditory”, “Ocular/nasal”, “Digestive/urogenital”, “Respiratory”, “Discomfort”, and “Other” disfunctions, and the assigned scores are 0 (absent), 0.5 (mild), and 1 (severe) for each of the 31 items. Another score employed is the Frailty Score that comprises five items: the running time (endurance), running speed (slowness), motor coordination, body weight, and grip strength (2/7 studies) [23,24], and is based on the previous frailty assessment developed for humans by Fried and co-workers [1]. Finally, another study [29] employed a modified version of the above-mentioned mouse frailty assessment designed by Whitehead et al., but readapted for an experimental autoimmune encephalomyelitis (EAE) mouse model. It contains 34 items, with “neuromusculoskeletal system/sensorimotor reflexes”, “paralysis and weakness”, and “ataxia/coordination” being added in comparison to the previous one.

Another important topic arising from this review is the gender difference, an argument that is still poorly highlighted in previous studies despite its importance in recent years. Focusing on this aspect, only two studies evaluated gender differences [20,24], while the others employed only one gender: males in 5/11 studies and females in 4/11 studies. It is difficult to draw a conclusion about gender differences using these two studies because the authors found contrasting results. In reality, hormones play a significant role in regulating various physiological processes in the body, including those related to muscle and connective tissue. For example, testosterone plays a crucial role in promoting muscle growth and strength, while estrogen influences muscle protein synthesis and can have a protective effect on muscle tissue. In addition, insulin can enhance muscle protein synthesis and contribute to muscle growth, and high cortisol levels over an extended period may contribute to muscle breakdown and hinder muscle growth. Growth hormone has anabolic effects on muscle tissue, promoting protein synthesis and supporting muscle growth, thyroid hormones can affect muscle function and play a role in maintaining connective tissue integrity, and insulin-like growth factor plays a role in promoting cell growth, including muscle cells [52,53].

The evaluation of gender differences in in vivo models is crucial for several reasons, as it can significantly impact the validity and applicability of the research findings and its translation into clinical practice. It allows for a more comprehensive understanding of health and disease and promotes gender-inclusive and equitable healthcare and medicine.

Frailty is a complex and multidimensional concept, and its presence or absence de-pends on a combination of biological, psychological, social, and environmental factors. Furthermore, frailty can vary over time and can be managed or prevented through appropriate interventions, such as exercise programs, lifestyle modifications, and appropriate medical care. Both researchers and clinicians should work collaboratively to bridge the gap between scientific knowledge and practical applications in the prevention and management of musculoskeletal frailty. This collaborative effort can lead to more effective strategies for improving the musculoskeletal health and overall well-being of older adults. For example, researchers should define and measure musculoskeletal frailty, conduct large-scale epidemiological studies, investigate the biomarkers associated with musculoskeletal frailty, implement longitudinal studies, explore and evaluate various interventions and treatments, and investigate the role of genetics and environmental factors. On the other hand, clinicians should develop and implement standardized screening tools to identify individuals at risk of musculoskeletal frailty, develop individualized care plans, prescribe tailored exercise programs, consider pharmacological interventions when appropriate, educate patients about the importance of a healthy lifestyle, and implement regular monitoring and follow-up assessments.

## 5. Conclusions

In vivo studies on musculoskeletal frailty are experimental models that are used to evaluate and better understand the mechanisms, causes, and potential treatments for musculoskeletal frailty, which is characterized by a loss of muscle mass, strength, and function, as well as a decline in bone density and quality. They provide a platform for controlled experimentation, reproducibility, and the investigation of complex physiological systems, ultimately benefiting both basic science research and potential clinical applications.

This article represents the first systematic review of in vivo models of frailty with a focus upon the musculoskeletal system. The review details the various models employed in research, as well as the corresponding techniques employed to evaluate levels of frailty. However, there is a need for additional studies to explore models that can better mimic human frailty and to explore gender differences, which is still a little-discussed topic. These studies evaluated frailty in four models: genetically modified or aged mice, injections of peptides, and with TS, resulting in musculoskeletal frailty that resembles that found in humans. To improve the translation of frailty models, FI—adapted from human tests—appears to be the most effective evaluation technique. As for treatments evaluated in in vivo models, AKG, MyMD-1, rapamycin, rSESN1, and LAMZ show promise in counteracting frailty.

An in vivo review of musculoskeletal frailty is integral to advancing both clinical practice and research efforts, offering insights that can guide effective interventions and improve outcomes for individuals affected by this condition. For clinicians, it is important for early detection and an accurate diagnosis, treatment planning and intervention, and the assessment of daily functioning and quality of life. For researchers, it is important for pathophysiology understanding and biomarker discovery.

## Figures and Tables

**Table 1:** Summary of the results of the included studies.

	In Vivo Model of Frailty	Evaluations	Main Results	Ref.
**Elderly models**	C57BL/6J female and male mice (18 mo old): Group 1: mice + standard-2918 diet; Group 2: mice + 2% *w*/*w* AKG supplemented on 2918 diet	FI; Survival; Indirect calorimetry (O_2_ consumption, CO_2_ production, whole-body composition); Treadmill exhaustion test (maximal speed and distance to exhaustion)	Group 2: ↑ survival and lifespan; ↓ FI, O_2_ consumption, CO_2_ production, energy expenditure	[20]
	C57BL/6J male mice: Group 1: mice (6 mo old); Group 2: mice (24 mo old); Group 3: mice (>28 mo old)	Functional test (endurance capacity, forelimb strenght, four limb strenght/endurance, balance, ccordination, gait speed, power generation, voluntary wheel running and activity rate, CFAB score); Body composition and muscle mass; Muscle contractile physiology of dorsiflexor torque	Groups 2, 3: ↓ grip, CFAB score, muscle contractile physiology; ↑ muscle mass than group 1. Group 3: ↓ inverted cling than group 1. Group 3: ↓ voluntary wheel running than groups 1, 2 Group 2: ↓ voluntary wheel running; ↑ body mass than group 1	[21]
	C57BL/6N male mice: Group 1: mice (3–8 mo old); Group 2: mice (13–23 mo old); Group 3: mice (27–36 mo old)	FI; Body composition (body fat, free body fluid, lean tissue content); Functional test (gait speed, tail height, forelimb strength); Micro-CT (Cr.Th, Tr.BMD, % fat around tibia, % lean around tibia); Metabolic assessment (natural walking gait speed); Indirect calorimetry (O_2_ consumption)	Group 3: ↑ FI, RER, % lean tissue around tibia; ↓ gait speed, forelimb muscle strength, locomotor activity, Cr.Th and Tr. BMD, body weight, % fat around tibia than groups 1, 2	[22]
	Group 1: C57BL/6 female mice (18–26 mo old); Group 2: G6PD-Tg female mice (18–26 mo old); Group 3: C57BL/6 female mice (34 mo old); Group 4: G6PD-Tg female mice (34 mo old)	Frailty score; Histology of gastrocnemius and tibialis anterior muscles (H&E staining); IHC of gastrocnemius and tibialis anterior muscles (eMHC); Metabolic assessment (EE, locomotory activity, RER); Body composition (BMD, lean mass, fat mass, fat in tissue)	Group 2: ↓ frailty score, RER; ↑ locomotory activity than group 1. Group 4: ↑ muscle fiber size than group 3	[23]
	C57BL/6J female and male mice (19 mo old): Group 1: mice + MyMD-1; Group 2: mice + high-dose (126 ppm) rapamycin; Group 3: mice + low-dose (14 ppm) rapamycin + metformin	FI; Health span assessment (body weight, grip strength, locomotor activity, motor coordination and endurance, learning and memory); Lifespan assessment	Groups 1, 2, 3: ↓ body weight, muscle strength; ↑ FI with age Group 1: ↑ health span characteristics, muscle strength; ↓ body weight loss, progression to frailty than group 3 Group 1: ↑ survival, lifespan than groups 2, 3	[24]
**Genetically** **modified models**	Group 1: C57BL/6J male mice (92 wks old); Group 2: IL10^tm/tm^ male mice (92 wks old)	^31^P MRS (PCr, P_i_, ADP, rate of ATP synthesis via CK (PCr → ATP), rate of ATP synthesis from P_i_ (P_i_ → ATP), free energy released from ATP hydrolysis (ΔG_∼ATP_)) in hind limb skeletal muscle	Group 2: ↓ PCr, ATP flux via CK, ATP synthesis from P_i_, free energy released from ATP hydrolysis; ↑ P_i_ than group 1	[25]
	Group 1: PolgA^(D257A/D257A)^ female mice (34 wks old); Group 2: PolgA^(+/+)^ female mice (34 wks old); Group 3: PolgA^(D257A/D257A)^ female mice (40 wks old); Group 4: PolgA^(+/+)^ female mice (40 wks old); Group 5: PolgA^(D257A/D257A)^ female mice (46 wks old); Group 6: PolgA^(+/+)^ female mice (46 wks old)	FI; Functional test (forelimb grip strength); Micro-CT of the right femur (BV/TV, Tb.Th, Tb.N, Tb.Sp, Ct.Ar/Tt.Ar, Ct.BV, Ct.MV, Ct.Ar, Tt.Ar, Ct.Th, Ps.Pm, Ec.Pm, AVD, length, BFR, BRR, MAR, MRR, MS, ES)	Groups 1, 3, 5: ↑ health deficits; ↓ bone mass, BVF, Tb.Th, Cr.Th, remodeling activities than groups 2, 4, 6 Groups 3, 5: ↑ FI; ↓ grip strength and concentric muscle forces than groups 4, 6	[26]
	Group 1: PolgA^(D257A/D257A)^ female mice (20–40 wks old); Group 2: PolgA^(+/+)^ female mice (20–40 wks old); Group 3: PolgA^(D257A/D257A)^ female mice (26–34 wks old); Group 4: PolgA^(+/+)^ female mice (26–34 wks old); Group 5: PolgA^(D257A/D257A)^ female mice (32–40 wks old); Group 6: PolgA^(+/+)^ female mice (32–40 wks old); Group 7: PolgA^(D257A/D257A)^ female mice (40–46 wks old); Group 8: PolgA^(+/+)^ female mice (40–46 wks old)	FI; Micro-CT of the 6th caudal vertebrae (BFR, BRR, MAR, MRR, MS, ES, BV/TV, Tb.Th, Tb.N., Ct.Ar/Tt.Ar, Ct.Th, Tb.Sp)	Groups 1, 3, 5, 7: ↓ BV/TV, Tb.Th, Tb.N, Ct.Ar/Tt.Ar, Ct.Th, BFR, BRR, MAR, MRR, MS; ↑ Tb.Sp, FI than groups 2, 4, 6, 8 Groups 7, 8: ↓ BV/TV, Tb.Th than groups 1, 2 Group 8: ↑ Tb.N, Ct.Ar/Tt.Ar; ↓ Tb.Sp, BRR, MRR than group 2	[27]
**Peptide** **injection model**	C57BL/6J male mice (16 mo old): Group 1: mice sham; Group 2: mice + CTX + PBS into tibialis anterior muscle/ quadriceps muscle; Group 3: mice + CTX + PBS + rSESN1 protein into tibialis anterior muscle/quadriceps muscle	Functional test (grip strength of forelimb and hind limb, motor coordination, maximal speed, time and distance to exhaustion)	Group 2: ↓ grip strength, physical endurance, maximal running time and running distance than groups 1, 3	[28]
	C57BL/6 female mice: Group 1: mice (3 mo old); Group 2: mice (3 mo old) + MOG peptide emulsified in CFA supplemented with heat-inactivated Mycobacterium tuberculosis; Group 3: mice (6 mo old) + MOG peptide emulsified in CFA supplemented with heat-inactivated Mycobacterium tuberculosis; Group 4: mice (12 mo old) + MOG peptide emulsified in CFA supplemented with heat-inactivated Mycobacterium tuberculosis	FI; Traditional 5-point clinical paralysis scale	Group 2: ↓ body weight; ↑ FI than group 1 Group 4: ↑ FI than groups 2, 3	[29]
**Tail-suspension model**	C57BL/6 J male mice (6 wks old): Group 1: TS model mice; Group 2: TS model mice + LAMZ (10 mg·kg^−1^ once a day for 14 days)	Micro-CT of femur (BV/TV, Tb.Sp, Tb.N, Tb.Th, BMC/TV, Cr.Th); Histology of proximal tibia (TRAP, toluidine blu/calcein staining); Histology of soleus muscle (H&E staining); Histomorphometry (Ob.Surf., Osteoid surf., BFR, Oc.N., Eroded surf., muscle fiber width); Functional test (fatigue-like behavior, travel distance, adjusted maximum muscle strength/g)	Group 1: ↓ muscle fiber width, travel distance, maximal muscle strength, BV/TV, Tb.N., Tb.Th., BMC/TV, Cr.Th., BFR, Ob.Surf., bone mass, osteoid surf.; ↑ episodes of fatigue-like behavior, Oc.N., eroded surf. than group 2	[30]

Abbreviations: Ref = references; mo = months; CFAB = Comprehensive Functional Assessment Battery; FI = frailty index; micro-CT = micro-computed tomography; Cr.Th = cortical thickness; Tr.BMD = trabecular bone mineral density; RER = respiratory exchange ratio; G6PD = Glucose 6-P dehydrogenase; H&E = haematoxylin and eosin; IHC = immunohistochemistry; eMHC = embryonic myosin heavy chain; EE = energy expenditure; BMD = bone mineral density; AKG = alpha-ketoglutarate; wks = weeks; IL = interleukin; MRS = magnetic resonance spectroscopy; PCr = phosphocreatine; Pi = inorganic phosphate; ADP = adenosine diphosphate; CK = creatine kinase; BV/TV = bone volume fraction; Tb.Th. = trabecular thickness; Tb.N = trabecular number; Tb.Sp. = trabecular spacing; Ct.Ar/Tt.Ar = cortical area fraction; Ct.BV = cortical bone volume; Ct.MV = cortical marrow volume; Ct.Ar = cortical area; Ct.Th = cortical thickness; AVD = apparent volume density; BFR = bone formation rate; BRR = bone resorption rate; MAR = mineral apposition rate; MRR = mineral resorption rate; MS = mineralizing surface; ES = eroded surface; CTX = cardiotoxin-induced; PBS = phosphate-buffered saline; rSESN1 = recombinant SESN1 protein; MOG = Myelin Oligodendrocyte Glycoprotein; CFA = Complete Freud’s Adjuvant; LAMZ = locamidazole; TS = tail suspension; BMC = bone marrow cells; TRAP = tartrate-resistant acid phosphatase; Ob = osteoblast; Oc = osteoclast; surf. = surface; ↑ = increase; ↓ = decrease.

**Table 2:** Frailty indices employed in animal models.

Frailty Index	Items	Sub-Items	Refs.
Mouse frailty assessment of Whitehead et al.	Integument	Alopecia; Loss of fur colour; Dermatitis; Loss of whiskers; Coat condition	[20,22,26,27]
	Physical/musculoskeletal	Tumors; Distended abdomen; Kyphosis; Tail stiffening; Gait disorders; Tremor; Forelimb grip strength; Body condition score	
	Vestibulocochlear/auditory	Vestibular disturbance; Hearing loss	
	Ocular/nasal	Cataracts; Corneal opacity; Eye discharge/swelling; Microphthalmia; Vision loss; Menace reflex; Nasal discharge	
	Digestive/urogenital	Malocclusions; Rectal prolapse; Vaginal/uterine/penile prolapse; Diarrhea	
	Respiratory	Breathing rate/depth	
	Discomfort	Mouse grimace scale; Piloerection	
	Other	Temperature; Weight	
Frailty score	Running time (endurance)	Derived from four paw hang and rotarod measures (seconds)	[23,24]
	Running speed (slowness)	Rotarod-training protocol (maximum speed)	
	Motor coordination	Voluntary wheel running (daily running distance)	
	Body weight	Low body weight	
	Grip strength	Four paw inverted hang (seconds to fall)	
Mouse frailty assessment adapted from Whitehead et al.	Integument	Alopecia; Dermatitis; Loss of whiskers	[29]
	Physical condition	Kyphosis; Tail condition; Gait; Body condition score; Distended abdomen	
	Neuromuscoskeletal system/ sensorimotor reflexes	Tremor; Hindlimb reflexology—foot “pinch”; Menace reflex	
	Paralysis and Weakness	Forelimb paralysis; Body posture; Nose down	
	Strength	Forelimb grip strength	
	Ataxia/coordination	Grid walk; Righting test; Splayed hind legs; Belly drag	
	Self-care and grooming	Coat condition	
	Vestibulocochlear system	Vestibular disturbance/head tilt	
	Auditory system	Hearing loss	
	Ocular system	Vision loss; Microphthalmia; Discharge/swollen/squinting	
	Nasal system	Nasal discharge	
	Digestive system	Diarrhoea	
	Urogenital system	Rectal prolapse; Vaginal/uterine prolapse	
	Respiratory	Breathing rate/depth	
	Discomfort	Mouse grimace scale; Piloerection; Temperature; Body weight	

## Data Availability

The data presented in this study are available on request from the corresponding author.

## References

[1] Fried L.P., Tangen C.M., Walston J., Newman A.B., Hirsch C., Gottdiener J., Seeman T., Tracy R., Kop W.J., Burke G. (2001). Frailty in older adults: Evidence for a phenotype. J. Gerontol. A Biol. Sci. Med. Sci..

[2] Rockwood K., Bergman H. (2021). FRAILTY: A report from the 3rd Joint Workshop of IAGG/WHO/SFGG, Athens, January 2012. Can. Geriatr. J..

[3] Clegg A., Young J., Iliffe S., Rikkert M.O., Rockwood K. (2013). Frailty in elderly people. Lancet.

[4] Franceschi C., Garagnani P., Parini P., Giuliani C., Santoro A. (2018). Inflammaging: A new immune-metabolic viewpoint for age-related diseases. Nat. Rev. Endocrinol..

[5] Vina J., Borras C., Gomez-Cabrera M.C. (2018). A free radical theory of frailty. Free Radic. Biol. Med..

[6] Collard R.M., Boter H., Schoevers R.A., Oude Voshaar R.C. (2012). Prevalence of frailty in communitydwelling older persons: A systematic review. J. Am. Geriatr. Soc..

[7] McPhee J.S., French D.P., Jackson D., Nazroo J., Pendleton N., Degens H. (2016). Physical activity in older age: Perspectives for healthy ageing and frailty. Biogerontology.

[8] McGuigan F.E., Bartosch P., Åkesson K.E. (2017). Musculoskeletal health and frailty. Best Pract. Res. Clin. Rheumatol..

[9] Milte R., Crotty M. (2014). Musculoskeletal health, frailty and functional decline. Best Pract. Res. Clin. Rheumatol..

[10] Frisoli A., Chaves P.H., Ingham S.J.M., Fried L.P. (2011). Severe osteopenia and osteoporosis, sarcopenia, and frailty status in community-dwelling older women: Results from the Women’s Health and Aging Study (WHAS) II. Bone.

[11] Fede C., Fan C., Pirri C., Petrelli L., Biz C., Porzionato A., Macchi V., De Caro R., Stecco C. (2022). The Effects of Aging on the Intramuscular Connective Tissue. Int. J. Mol. Sci..

[12] Fan C., Pirri C., Fede C., Guidolin D., Biz C., Petrelli L., Porzionato A., Macchi V., De Caro R., Stecco C. (2021). Age-Related Alterations of Hyaluronan and Collagen in Extracellular Matrix of the Muscle Spindles. J. Clin. Med..

[13] Pavan P., Monti E., Bondí M., Fan C., Stecco C., Narici M., Reggiani C., Marcucci L. (2020). Alterations of Extracellular Matrix Mechanical Properties Contribute to Age-Related Functional Impairment of Human Skeletal Muscles. Int. J. Mol. Sci..

[14] Buta B.J., Walston J.D., Godino J.G., Park M., Kalyani R.R., Xue Q., Bandeen-Roche K., Varadhan R. (2016). Frailty assessment instruments: Systematic characterization of the uses and contexts of highlycited instruments. Ageing Res. Rev..

[15] Searle S.D., Mitnitski A., Gahbauer E.A., Gill T.M., Rockwood K. (2008). A standard procedure for creating a frailty index. BMC Geriatr..

[16] Whitehead J.C., Hildebrand B.A., Sun M., Rockwood M.R., Rose R.A., Rockwood K., Howlett S.E. (2014). A clinical frailty index in aging mice: Comparisons with frailty index data in humans. J. Gerontol. A Biol. Sci. Med. Sci..

[17] Kane A.E., Hilmer S.N., Mach J., Mitchell S.J., de Cabo R., Howlett S.E. (2016). Animal models of frailty: Current applications in clinical research. Clin. Interv. Aging.

[18] von Zglinicki T., Varela-Nieto I., Brites D., Karagianni N., Ortolano S., Georgopoulos S., Cardoso A.L., Novella S., Lepperdinger G., Trendelenburg A.U. (2016). Frailty in mouse ageing: A conceptual approach. Mech. Ageing Dev..

[19] Banga S., Heinze-Milne S.D., Howlett S.E. (2019). Rodent models of frailty and their application in preclinical research. Mech. Ageing Dev..

[20] Asadi Shahmirzadi A., Edgar D., Liao C.Y., Hsu Y.M., Lucanic M., Asadi Shahmirzadi A., Wiley C.D., Gan G., Kim D.E., Kasler H.G. (2020). Alpha-Ketoglutarate, an Endogenous Metabolite, Extends Lifespan and Compresses Morbidity in Aging Mice. Cell Metab..

[21] Graber T.G., Maroto R., Fry C.S., Brightwell C.R., Rasmussen B.B. (2021). Measuring Exercise Capacity and Physical Function in Adult and Older Mice. J. Gerontol. A Biol. Sci. Med. Sci..

[22] Petr M.A., Alfaras I., Krawcyzk M., Bair W.N., Mitchell S.J., Morrell C.H., Studenski S.A., Price N.L., Fishbein K.W., Spencer R.G. (2021). A cross-sectional study of functional and metabolic changes during aging through the lifespan in male mice. eLife.

[23] Arc-Chagnaud C., Salvador-Pascual A., Garcia-Dominguez E., Olaso-Gonzalez G., Correas A.G., Serna E., Brioche T., Chopard A., Fernandez-Marcos P.J., Serrano M. (2021). Glucose 6-P dehydrogenase delays the onset of frailty by protecting against muscle damage. J. Cachexia Sarcopenia Muscle.

[24] Sabini E., O’Mahony A., Caturegli P. (2023). MyMD-1 Improves Health Span and Prolongs Life Span in Old Mice: A Noninferiority Study to Rapamycin. Gerontol. A Biol. Sci. Med. Sci..

[25] Akki A., Yang H., Gupta A., Chacko V.P., Yano T., Leppo M.K., Steenbergen C., Walston J., Weiss R.G. (2014). Skeletal muscle ATP kinetics are impaired in frail mice. Age.

[26] Scheuren A.C., Kuhn G.A., Müller R. (2020). Effects of long-term in vivo micro-CT imaging on hallmarks of osteopenia and frailty in aging mice. PLoS ONE.

[27] Scheuren A.C., D’Hulst G., Kuhn G.A., Masschelein E., Wehrle E., De Bock K., Müller R. (2020). Hallmarks of frailty and osteosarcopenia in prematurely aged PolgA(D257A/D257A) mice. J. Cachexia Sarcopenia Muscle.

[28] Jing Y., Zuo Y., Sun L., Yu Z.R., Ma S., Hu H., Zhao Q., Huang D., Zhang W., Izpisua Belmonte J.C. (2023). SESN1 is a FOXO3 effector that counteracts human skeletal muscle ageing. Cell Prolif..

[29] Ribeiro A.R., Barros C., Barateiro A., Howlett S.E., Fernandes A. (2022). Improved Assessment of Overall Health in Variably Aged Murine Models of Multiple Sclerosis With a Novel Frailty Index Tool. J. Gerontol. A Biol. Sci. Med. Sci..

[30] Ono T., Denda R., Tsukahara Y., Nakamura T., Okamoto K., Takayanagi H., Nakashima T. (2022). Simultaneous augmentation of muscle and bone by locomomimetism through calcium-PGC-1α signaling. Bone Res..

[31] Hooijmans C.R., Rovers M.M., de Vries R.B., Leenaars M., Ritskes-Hoitinga M., Langendam M.W. (2014). SYRCLE’s risk of bias tool for animal studies. BMC Med. Res. Method.

[32] Joseph B., Phelan H., Hassan A., Orouji Jokar T., O’Keeffe T., Azim A., Gries L., Kulvatunyou N., Latifi R., Rhee P. (2016). The impact of frailty on failure-torescue in geriatric trauma patients: A prospective study. J. Trauma Acute Care Surg..

[33] Kim S.W., Han H.S., Jung H.W., Kim K.I., Hwang D.W., Kang S.B., Kim C.H. (2014). Multidimensional frailty score for the prediction of postoperative mortality risk. JAMA Surg..

[34] Kositsawat J., Duque G., Kirk B. (2021). Nutrients with anabolic/anticatabolic, antioxidant, and anti-inflammatory properties: Targeting the biological mechanisms of aging to support musculoskeletal health. Exp. Gerontol..

[35] Miller R.A., Harper J.M., Galecki A., Burke D.T. (2002). Big mice die young: Early life body weight predicts longevity in genetically heterogeneous mice. Aging Cell.

[36] Mitchell S.J., Bernier M., Mattison J.A., Aon M.A., Kaiser T.A., Anson R.M., Ikeno Y., Anderson R.M., Ingram D.K., de Cabo R. (2019). Daily fasting improves health and survival in male mice independent of diet composition and calories. Cell Metab..

[37] TeSlaa T., Chaikovsky A.C., Lipchina I., Escobar S.L., Hochedlinger K., Huang J., Graeber T.G., Braas D., Teitell M.A. (2016). α-Ketoglutarate Accelerates the Initial Differentiation of Primed Human Pluripotent Stem Cells. Cell Metab..

[38] Su Y., Wang T., Wu N., Li D., Fan X., Xu Z., Mishra S.K., Yang M. (2019). Alpha-ketoglutarate extends Drosophila lifespan by inhibiting mTOR and activating AMPK. Aging.

[39] Di Dalmazi G., Chalan P., Caturegli P. (2019). MYMD-1, a novel immunometabolic regulator, ameliorates autoimmune thyroiditis via suppression of Th1 responses and TNF-α release. J. Immunol..

[40] Berg E.L., Polokoff M.A., O’Mahony A., Nguyen D., Li X. (2015). Elucidating mechanisms of toxicity using phenotypic data from primary human cell systems―a chemical biology approach for thrombosis-related side effects. Int. J. Mol. Sci..

[41] Nóbrega-Pereira S., Fernandez-Marcos P.J., Brioche T., Gomez-Cabrera M.C., Salvador-Pascual A., Flores J.M., Viña J., Serrano M. (2016). G6PD protects from oxidative damage and improves healthspan in mice. Nat. Commun..

[42] Cappellini M.D., Fiorelli G. (2008). Glucose-6-phosphate dehydrogenase deficiency. Lancet.

[43] Kujoth G.C., Hiona A., Pugh T.D., Someya S., Panzer K., Wohlgemuth S.E., Hofer T., Seo A.Y., Sullivan R., Jobling W.A. (2005). Mitochondrial DNA mutations, oxidative stress, and apoptosis in mammalian aging. Science.

[44] Trifunovic A., Wredenberg A., Falkenberg M., Spelbrink J.N., Rovio A.T., Bruder C.E., Bohlooly-Y M., Gidlöf S., Oldfors A., Wibom R. (2004). Premature ageing in mice expressing defective mitochondrial DNA polymerase. Nature.

[45] Walston J., Fedarko N., Yang H., Leng S., Beamer B., Espinoza S., Lipton A., Zheng H., Becker K. (2008). The physical and biological characterization of a frail mouse model. J. Gerontol. Ser. A Biol. Sci. Med. Sci..

[46] Ko F., Yu Q., Xue Q.L., Yao W., Brayton C., Yang H., Fedarko N., Walston J. (2012). Inflammation and mortality in a frail mouse model. Age.

[47] Swanson C.W., Richmond S.B., Sharp B.E., Fling B.W. (2021). Middle-age people with multiple sclerosis demonstrate similar mobility characteristics to neurotypical older adults. Mult. Scler. Relat. Disord..

[48] Kim M., Kowalsky A.H., Lee J.H. (2021). Sestrins in physiological stress responses. Annu. Rev. Physiol..

[49] Chen Y., Huang T., Yu Z., Yu Q., Wang Y., Hu J., Shi J., Yang G. (2022). The functions and roles of sestrins in regulating human diseases. Cell Mol. Biol. Lett..

[50] Dent E., Kowal P., Hoogendijk E.O. (2016). Frailty measurement in research and clinical practice: A review. Europ. J. Int. Med..

[51] de Vries N.M., Staal J.B., van Ravensberg C.D., Hobbelen J.S., Olde Rikkert M.G., Nijhuis-van der Sanden M.W. (2011). Outcome instruments to measure frailty: A systematic review. Ageing Res. Rev..

[52] Fink J., Schoenfeld B.J., Nakazato K. (2018). The role of hormones in muscle hypertrophy. Phys. Sportsmed..

[53] Boyan B.D., Hart D.A., Enoka M., Nicolella D.P., Resnick E., Berkley K.J., Sluka K.A., Kwoh C.K., Tosi L.L., O’Connor M.I. (2013). Hormonal modulation of connective tissue homeostasis and sex differences in risk for osteoarthritis of the knee. Biol. Sex Differ..

